# Artificial intelligence in virtual fracture clinics: a systematic review of imaging and clinical-text tools

**DOI:** 10.1186/s13018-025-06656-5

**Published:** 2026-02-06

**Authors:** Tenghis Sukhbaatar, Andrew Davies, Aran Koye, Mohamed Hashem, Sivan Sivaloganathan

**Affiliations:** 1 Imperial College London, London, UK; 2 Chelsea and Westminster Hospital NHS Foundation Trust, London, UK; 3 Frimley Health NHS Foundation Trust, Frimley, UK

## Abstract

**Background:**

Virtual fracture clinics (VFCs) are a well-established component of acute orthopedic management pathways. Artificial intelligence (AI) healthcare tools are increasingly sophisticated and have the potential to disrupt current practices. The aim of this review was to determine the opportunities, performance and readiness of AI systems that integrate clinical-text and imaging data for the triage or management of patients in VFCs.

**Methods:**

A search of MEDLINE and Embase was performed between January 2010 and July 2025. The review included primary research studies investigating AI for fracture detection via X-rays and natural language processing (NLP) models for clinical documentation. A random-effects meta-analysis was conducted to calculate pooled sensitivity and specificity, stratified by anatomical region and developer type (commercial vs. researcher-developed).

**Results:**

We included 54 studies: 52 imaging/X-ray studies and 2 NLP/clinical-text studies. Among the imaging studies, 13 evaluated commercial tools, and 39 evaluated researcher-developed models. There were 2 NLP models, both of which interpreted radiology reports rather than text summaries of clinical assessments. No studies that included the use of NLP models in acute orthopedic care could be found. A meta-analysis of commercial tools (*n* = 11) demonstrated a pooled sensitivity across both multiregional “Limb” tools of 92.58% (95% CI 90.61–94.17%) and anatomy-specific “Wrist” tools of 89.95% (95% CI 72.18–96.86%). Wrist-specific commercial tools demonstrated higher specificity (96.80%; 95% CI 90.12–99.01%) compared to general limb tools (89.69%; 95% CI 84.02–93.51%), suggesting that anatomical targeting may reduce the number of false positives. Researcher-developed models (*n* = 32) often reported superior metrics for sensitivity compared to the sensitivity of commercial tools.

**Conclusions:**

VFCs require the integration of information from imaging and patient records. Multiple image interpretation tools are available with high performance in fracture identification. The development and integration of NLP tools to interpret clinical documentation from emergency departments and urgent care centers are necessary for AI-VFC.

**Supplementary Information:**

The online version contains supplementary material available at 10.1186/s13018-025-06656-5.

## Background

Virtual fracture clinics (VFCs) are now embedded across UK orthopedic services and have demonstrated improvements in access, throughput and costs while maintaining care standards [1–3]. The expansion of artificial intelligence (AI) healthcare tools may provide an opportunity to further improve the efficiency of acute orthopedic care.

There are two main components of the VFC process: the review of radiographs combined with emergency department (ED) documentation and urgent care center documentation to inform clinical decisions. There is emerging evidence that integrating AI can help address both tasks [4–6].

###  How VFCs could utilize AI?

- X-ray review at scale. Imaging AI may identify whether a fracture, dislocation, or alternative injury is present (and where), separate common mimics, and indicate uncertainty clearly enough to support safe triage (case-level “fracture likely/unlikely,” region-specific flags, and a simple visual cue).
- Clinical decision-making from text. ED notes capture mechanisms, examination findings, initial management, and safety netting. An NLP system may extract key facts and map patients to locally agreed pathways (e.g., “virtual review only,” “face-to-face within 72 h,” “immediate escalation”). NLP can support decision-making alongside X-ray interpretation and help propose a plan [4, 6–8].

The diagnostic performance for the detection of some fractures on radiographs is comparable to that of clinicians (typical pooled sensitivity/specificity of ~ 0.90–0.92), albeit with heterogeneity and frequent study bias; performance varies by anatomical region [5, 9–11].

In January 2025, the NICE (National Institute for Health and Care Excellence) EVA (Early Value Assessment) issued conditional (evidence-generation) recommendations for the use of AI to help detect fractures via urgent-care X-rays. The technologies listed include BoneView (Gleamer), Rayvolve (AZmed), RBfracture (Radiobotics), TechCare Alert (Milvue), and qMSK (Qure.ai), which are all intended as decision aids integrated with picture archiving and communication systems (PACSs). The guidance emphasizes governance, local calibration, and clear protocols for disagreements between AI and clinicians [4].

ED triage documentation is largely unstructured. Reviews of NLP at triage show that models using free text (alone or combined with structured variables) improve the prediction of decisions such as the need for admission or severity of illness versus structured data alone, supporting the concept that text-driven decisions could be applied to VFC referrals and mapping patients to the right pathway [6–8].

Given the rapid development of AI healthcare programs, no prior review has examined the potential for the integration of fracture detection and NLP tools into the VFC pathway. The aim of this review was to explore the tools available for an AI virtual fracture clinic.

## Methods

### Protocol and registration

The review protocol was registered on the International Prospective Register of Systematic Reviews (PROSPERO) database (PROSPERO ID: CRD420251057551; registration date: 21/05/2025), and the report was aligned with the Preferred Reporting Items for Systematic Reviews and Meta-Analyses (PRISMA) 2020 checklist (see appendix).

### Search strategy

The MEDLINE (Ovid) and Embase (Ovid) databases were searched from Jan 2010 to July 2025, with the final search run on 30 July 2025. The search strategy combined headings and free-text terms for fractures, AI, triage, and radiography (see appendix).

### Eligibility criteria

Patients of any age who presented with suspected acute musculoskeletal trauma were included. Interventions included AI-enabled tools supporting fracture diagnosis via radiographs, decision support/triage (including the NLP of clinical text), or multimodal tools used within virtual care. All study designs were included.

We excluded fractures that would not be referred to a virtual fracture clinic (e.g., hip fracture), AI applied only to CT/MRI, non-MSK conditions, studies focused exclusively beyond triage (e.g., surgical planning/rehab), AI algorithm development without clinical validation, non-AI decision support, telemedicine without an AI element, editorials/opinions, animal/cadaver studies, and studies not in English.

### Study selection

Screening for eligibility was performed in duplicate by two independent reviewers (T.S. and A.K.), and disagreements were resolved by discussion with a third independent reviewer (A.R.D.). The search results were organized via Covidence systematic review management software. Duplicates were removed, and articles were selected according to the inclusion criteria.

### Data collection and management

Two reviewers independently assessed the full texts and extracted the data via a preagreed-upon data collection form. The extracted data included the setting, population, study design, AI tool/company/product/version, dataset, regulatory status, anatomical sites used, workflow/economic endpoints, and integration/governance. Disagreements were resolved by consensus or by a third reviewer. Where available, we extracted data on tool performance. The prespecified subgroups included the anatomical region (e.g., wrist, hand, ankle/foot, elbow/shoulder), model scope (region-specific vs. multiregion), and developer type (commercial vs. researcher-developed). When studies reported multiple compatible results, we applied prespecified rules: prefer external over internal test sets; prefer per-image over per-patient metrics; and report stand-alone AI results when available.

### Statistical analysis

We conducted a random-effects meta-analysis on a subset of included studies where full contingency tables (true positive, false negative, true negative, false positive) were available or could be derived. We performed separate analyses for commercial tools and researcher-developed models to account for the distinct differences in validation rigor and study design between these groups.

The analysis was stratified by anatomical region (e.g., limbs, wrist, ankle, elbow) to assess performance variability across different clinical tasks. Pooled estimates for sensitivity and specificity were calculated via a generalized linear mixed model (GLMM) with logit transformation. We applied the Hartung-Knapp adjustment to construct 95% confidence intervals (CIs), as this method provides more robust error estimation for meta-analyses with varying study sizes [12]. Heterogeneity was assessed via the I^2^ statistic and X^2^ test. All analyses were performed using the meta package in R (version 4.5.2).

### Risk of bias and applicability

The Quality Assessment of Diagnostic Accuracy Studies 2 (QUADAS-2) [13] recommended by the Cochrane Collaboration was used to evaluate the risk of bias. A modified version of the suggested list of questions was created for each domain adapted for this systematic review, and the complete list of questions is provided in the appendix. Four domains: patient selection, the index test, the reference standard, and flow and timing, were qualified with the support of the signaling questions as ‘low concern’, ‘high concern’ or ‘unclear’ for risk of bias and applicability. The assessments were performed independently by two authors (T.S. and A.K.). If the two researchers could not reach a consensus, the senior author was consulted.

## Results

**Fig. 1:**
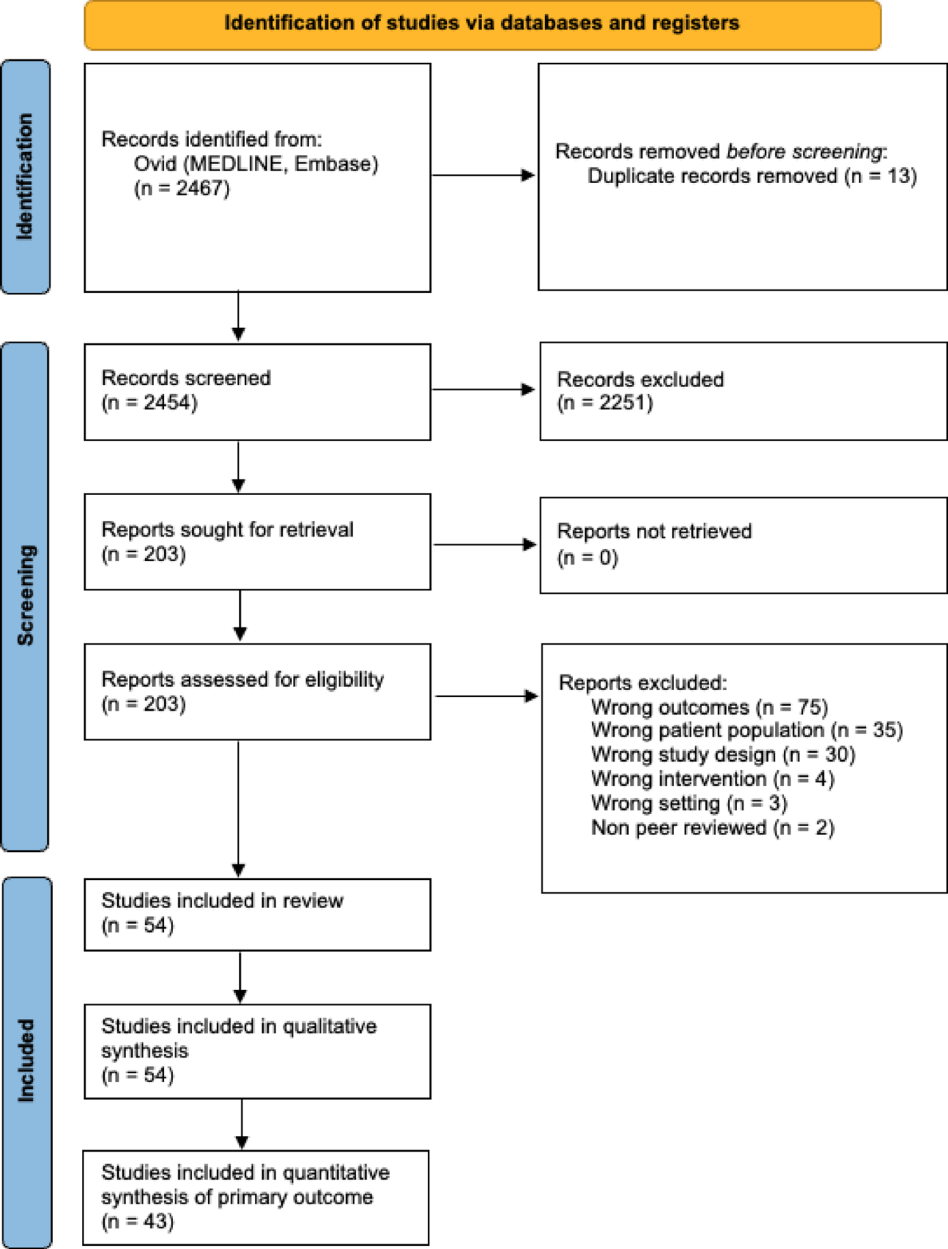
Reporting items for systematic reviews PRISMA 2020 flowchart showing studies selected for review

We identified 2467 studies, of which 12 were duplicates. Fifty-four studies met the inclusion criteria: 52 studies focused on X-ray imaging, and 2 explored NLP/clinical-text analysis. Imaging studies were split into commercial tools (*n* = 13; products: BoneView, RBfracture, Rayvolve, MediAI-FX, SmartUrgence) and researcher-developed (no brand) models (*n* = 39). The PRISMA flow diagram is shown in Fig. 1.

### Commercial AI tools for fracture detection

Thirteen studies evaluated commercially available AI systems designed for clinical deployment (Table 1). These tools represent fracture detection technologies, several of which have obtained regulatory clearance and are progressing through NHS implementation pathways, as outlined in NICE guidance. A detailed overview of their specifications is included (Table 2).

BoneView (Gleamer) was the most frequently evaluated system and was used in seven studies. This CNN-based tool provides both case-level fracture probability scores and overlays that highlight suspected fracture regions on radiographs. Dislocations, joint effusions and bone lesions are also captured. The system processes the appendicular skeleton and rib radiographs, which are integrated directly with PACS. Studies have evaluated BoneView across diverse clinical settings, from emergency departments to outpatient clinics, with training datasets ranging from 2456 to 312,602 radiographs [14–21].

RBfracture (Radiobotics) was evaluated in two large-scale studies. This system generates case-level alerts with anatomical localization for appendicular fractures. The tool employs deep learning architectures trained on approximately 320,000 radiographs and has been tested in urgent care workflows where it flags high-priority cases for expedited review [22, 23].

Rayvolve (AZmed) uses a CNN to detect fractures with bounding box localization and confidence scoring. Two studies examined its performance in multisite emergency department settings, with one comparative study evaluating it alongside BoneView and SmartUrgence on identical datasets of 1500 radiographs [16, 24].

Milvue is a relatively recent commercial entrant. In one large-scale retrospective study, a Milvue AI software package (product name not specified) was trained on > 1.26 million radiographs and evaluated for automated alerting and case prioritization in emergency radiography workflows [25]. In a separate study that explicitly named the product, SmartUrgence (Milvue), a tool designed to provide instant triage/primary findings and support worklist prioritization in emergency and routine radiography, was evaluated [16].

Another commercial system evaluated was MediAI-FX (Crescom, South Korea), a decision-support model; the included studies reported variation in output display, integration approach, age indications, and specific wrist radiograph targets (Table 3) [26].

**Table 1:** Performance of commercial AI systems for fracture detection on radiographs

Reference	AI Model	Product	Anatomical region	Radiographs training/testing	AUC	Accuracy (%)	Specificity (%)	Sensitivity (%)
Hayashi et al. 2022 [14]	CNN	BoneView	Limbs	312,602/300	0.93	–	90	91.3
Duron et al. 2021 [15]	CNN	BoneView	Limbs	60,170/2441	0.94	–	–	–
Bousson et al. 2023 [16]	CNN	Rayvolve	Limbs	–/1500	–	71	70.4	92.6
	CNN	SmartUrgence	Limbs	–/1500	–	90.1	92.5	90.2
	CNN	BoneView	Limbs	–/1500	–	88.8	90.5	91.3
Guermazi et al. 2022 [17]	CNN	BoneView	Limbs	60,170/480	0.93	–	88	88
Dell’Aria et al. 2024 [18]	CNN	BoneView	Limbs	–/101	0.92	–	89.29	95.45
Cohen et al. 2023 [19]	CNN	BoneView	Wrist	60,170/1917	–	–	96	83
Russe et al. 2024 [20]	CNN	BoneView	Wrist	2057/400	–	95	95	95
Ramadanov et al. 2025 [21]	CNN	BoneView	Wrist	500,000/1114	–	95.33	98.35	90.85
Bachmann et al. 2024 [22]	CNN	RBfracture	Limbs	> 320,000/340	0.94	–	88	92
Ziegner et al. 2025 [23]	CNN	RBfracture	Limbs	–/1672	–	87	83	92
Dupuis et al. 2022 [24]	CNN	Rayvolve	Limbs	21,138/2634	–	92.6	91.2	95.7
Diaz et al. 2025 [25]	CNN	AI software (Milvue)	Limbs	1,262,467/792	0.97	–	97.6	95.8
Lee et al. 2023 [26]	RetinaNet + NasNet	MediAI-FX	Wrist	4432/186	–	–	–	–

**Table 2:** Overview of commercially available NICE-approved artificial intelligence solutions for fracture and injury detection [4]

AI technology (manufacturer)	CE marking	Regions covered	Population	Other pathologies detected	Intended use & output
BoneView (Gleamer)	Class IIa	Appendicular skeleton, ribs and thoracic-lumbar spine	2 years and over	Dislocation, effusion, bone lesion	Computer assisted detection for musculoskeletal radiographs; outputs bounding boxes/visual overlays.
Rayvolve (AZmed)	Class IIa	Appendicular skeleton and ribs	No age limit	Dislocation, joint effusion, and chest pathologies (pneumothorax, cardiomegaly, pleural effusion, pulmonary edema, consolidation, nodule)	Aided diagnosis; outputs regions/boxes to support the reader.
RBfracture (Radiobotics)	Class IIa	Appendicular skeleton and ribs	2 years and over	Effusion of the knee and elbow, lipohaemarthrosis of the knee	Decision-support/assist with visual outputs for clinicians.
TechCare Alert (Milvue)	Class IIa	Appendicular skeleton and ribs	No age limit	Dislocation, elbow joint effusion, pleural effusion, pulmonary opacity, pulmonary nodule, pneumothorax	Assistive triage; designed to support prioritization and diagnostic quality for radiologists/ED teams.

**Table 3:** Overview of commercially available artificial intelligence (AI) tools for fracture detection that have not received NICE approval

AI technology (manufacturer)	CE marking	Regions covered	Population	Other pathologies detected	Intended use & outputs
SmartUrgences (Milvue)	Class IIa	Appendicular skeleton, and ribs	Adults & pediatrics	Joint dislocation, elbow joint effusion, rib fracture, pleural effusion, pulmonary opacity, pulmonary nodule, pneumothorax	Assistive triage; supports workflow prioritization
MediAI-FX (Crescom)	MFDS (Korea) approval; CE marking not stated	Wrist—distal radius, ulnar styloid, scaphoid	Adults (94.6% in clinical validation study)	–	Assistive wrist-fracture detection; provides fracture probability/localization to support quick
qMSK (Qure.ai)*	Class IIb	Appendicular skeleton and ribs	2 years and over	–	Assistive musculoskeletal triage; preread bounding boxes, worklist prioritization

*More research is needed on qMSK (not captured in this review) to help healthcare professionals detect fractures on X-rays of adults in urgent care before it can be used in the NHS [4]

### Researcher-developed models

Thirty-nine studies described AI models developed by research teams, providing insights into emerging technical approaches and potential future commercial tools (Table 4). These studies explored diverse deep learning architectures and implementation strategies.

Technical Architectures: Research teams have employed various CNN architectures [27–35], including ResNet [36–40], DenseNet [41–46], YOLO variants [47–50, 65], Inception [51, 52], and custom CNN designs [53–59]. Several studies have incorporated region-based convolutional neural networks (R-CNNs) for precise fracture localization, whereas others have focused on classification tasks alone [50, 60–64].

Anatomical Focus: Studies distributed across anatomical regions with notable clustering around wrist fractures (20 studies) [31–34, 40–42, 44, 48–50, 52, 54, 55, 58, 59, 62–65], reflecting the high incidence and diagnostic challenge of these injuries in emergency settings. Multiregion appendicular models were used in 5 studies [46, 47, 56, 60, 61], while specific focus areas included the ankle [35, 51], shoulder [29, 30, 38, 45], elbow [27, 37, 39, 43], foot [28, 57], and hand [36].

Training Approaches: Dataset sizes vary considerably, from focused single-center collections of 280 radiographs to large multi-institutional datasets exceeding 700,000 images. Most models have undergone internal validation, although external validation on completely independent datasets remains uncommon.

Output Formats: Models generate various output types, including binary classification (fracture present/absent), multiclass classification (fracture type/location), probability scores, visual overlays (heatmaps, bounding boxes), and region-specific alerts. Several studies have explored ensemble methods that combine multiple model predictions.

**Table 4:** Overview of academic and researcher-developed AI models for fracture detection on radiographs

References	AI Model	Anatomical Region	Radiographs Training/Testing	AUC	Accuracy (%)	Specificity (%)	Sensitivity (%)
Ye et al. 2025 [27]	CNN	Elbow	1000/180	–	90.5	92	89
Kim et al. 2023 [28]	CNN	Foot	934/165	0.95	86.1	92.2	78.7
Alzubaidi et al. 2024 [29]	CNN	Shoulder	8379/563	–	99.2	98.9	99.6
Magneli et al. 2023 [30]	CNN	Shoulder	6221/1257	0.92	–	89	83
Breu et al. 2024 [31]	CNN	Wrist	19,783/200	0.97	93.5	91	96
Yang et al. 2024 [32]	CNN	Wrist	140/35	–	89.94	92	87.33
Anttila et al. 2023 [33]	CNN	Wrist	3399/386	0.97	92	80	97
Langerhuizen et al. 2020 [34]	CNN	Wrist	180/100	0.77	72	60	84
Kitamura et al. 2019 [35]	CNN	Ankle	1441/240	–	81	83	80
Axenhus et al. 2025 [36]	ResNet	Hand	7100/327	0.79	71	63	80
Choi et al. 2020 [37]	ResNet models	Elbow	2024/516	0.99	92.6	92.2	93.9
Chung et al. 2018 [38]	ResNet-152	Shoulder	40,000/181	0.99	96	0.97	0.99
Kekatpure et al. 2024 [39]	ResNet18	Elbow	3944/987	0.79	69.14	95.89	61.49
Ozkaya et al. 2022 [40]	ResNet50	Wrist	203/100	0.84	84	92	76
Hendrix et al. 2021 [41]	DenseNet-121	Wrist	3000/190	0.86	–	84	78
Kim et al. 2021 [42]	DenseNet-161	Wrist	8994/990	0.96	90.3	90.3	90.3
Li et al. 2024 [43]	DenseNet-201	Elbow	1096/274	0.89	90.5	94.7	81.4
Gan et al. 2024 [44]	DenseNet121	Wrist	1344/448	0.94	85.7	83	90.8
Dasegowda et al. 2024 [45]	DenseNet201	Shoulder	2151/1666	0.95	88	87	90
Aldhyani et al. 2024 [46]	DenseNet201	Limbs	7406/2115	0.99	97.35	97.06	97.78
Wei et al. 2025 [47]	YOLOv11	Limbs	10,966/2350	0.97	95.14	95.15	95.14
Binh et al. 2024 [48]	YOLOv4	Wrist	7006/88	> 0.80	–	–	–
Ahmed et al. 2024 [49]	YOLOv8m	Wrist	15,245/1016	–	92	95	93
Thian et al. 2019 [50]	R-CNN	Wrist	6515/524	0.92	88.9	82.5	95.7
Ashkani-Esfahani et al. 2022 [51]	Inception V3	Ankle	1260/420	0.95	92	94	91
Kyung et al. 2024 [52]	Inception V3	Wrist	12,196/4065	0.97	93.2	89.1	95
Cheng et al. 2023 [53]	DCNN	Ankle	2702/200	0.92	87	90	84
Yoon et al. 2021 [54]	DCNN	Wrist	8356/2305	–	78.5	66	97.2
Rashid et al. 2023 [55]	DCNN	Wrist	–/–	–	88.28	82.93	90
Jones et al. 2020 [56]	DL Model	Limbs	715,343/16,019	0.97	–	80	95.2
Yu et al. 2025 [57]	MTL-DlinkNet	Foot	–/446	0.99	95.7	–	94.4
Ureten et al. 2022 [58]	VGG-16	Wrist	697/135	–	93.3	90.3	96.8
Bluthgen et al. 2020 [59]	ViDi Suite 2.0	Wrist	524/200	0.87	83	86	80
Franco et al. 2024 [60]	R-CNN	Limbs	5295/600	–	84	76.7	91.3
Zech et al. 2024 [61]	R-CNN	Upper Limbs	48,807/240	0.89	–	88.3	90
Zech et al. 2023 [62]	R-CNN	Wrist	229/125	0.92	88	89	88
Gan et al. 2019 [63]	R-CNN	Wrist	2040/300	0.96	93	96	90
Zhang et al. 2023 [64]	R-CNN	Wrist	4579/978	–	97.55	96.73	98.36
Li et al. 2023 [65]	YOLOv3	Wrist	930/209	0.92	88	94	82

### Natural language processing systems

Only two studies were captured in our review, which examined NLP applications for fracture-related clinical text, highlighting a significant evidence gap in multimodal AI approaches (Table 5). The first study, from the Netherlands, developed an NLP system to classify radiology reports for upper- and lower-body fractures, achieving high classification accuracy when tested on 1377 reports [66].

**Table 5:** Overview of academic and researcher-developed AI models for fracture detection on radiographs

Reference	AI Model	Brand name	Anatomical region	Radiographs training/testing	AUC	Accuracy	Specificity	Sensitivity
Olthof et al. 2021 [66]	NLP	–	Limbs	2469/1377	99	96	98	95
Zech et al. 2023 [67]	CNN + NLP	–	Upper Limbs	48,007/4057	96	89.7	90.8	88.7

The second study combined CNN image analysis with NLP text processing, creating a hybrid system that analyzed both the radiographs and the documentation of the radiographs. This multimodal approach processed 48,007 training images alongside corresponding radiograph text, demonstrating the potential for integrated decision support [67].

### Meta-analyses: commercial AI tools

**Fig. 2:**
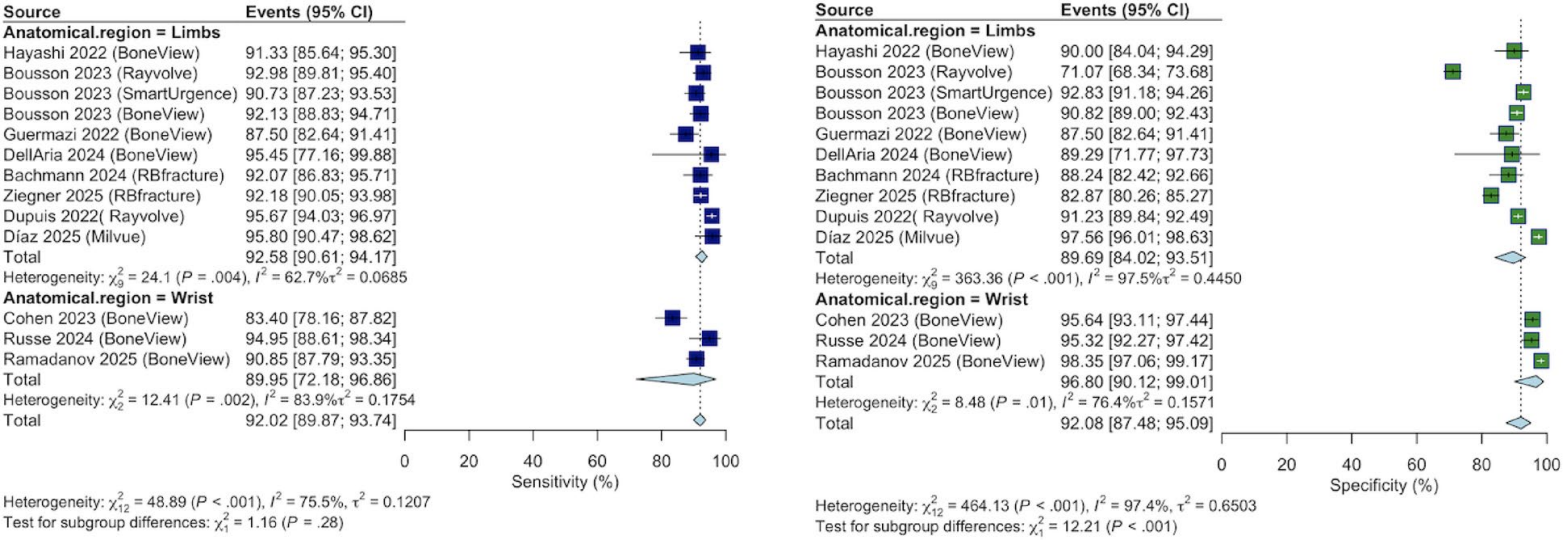
Forest plots of diagnostic accuracy for commercial AI tools, stratified by anatomical scope (Limbs vs. Wrist)

A random-effects meta-analysis was performed on 11 of the 13 commercial tool studies (Fig. 2). Two studies, Duron et al. 2021 [15] and Lee et al. 2023 [26], were excluded from the pooled analysis, as both had insufficient contingency table data for sensitivity/specificity calculations.

The diagnostic performance of commercial tools included the following:

Sensitivity: The overall pooled sensitivity was 92.02% (95% CI 89.87–93.74) with substantial heterogeneity (I^2^ =75.5%). According to the anatomical subgroup, the pooled sensitivity was 92.58% (90.61–94.17) for the limbs and 89.95% (72.18–96.86) for the wrist.

Specificity: The overall pooled specificity was 92.08% (95% CI 87.48–95.09) with very high heterogeneity (I^2^= 97.4%). Wrist-specific tools showed higher pooled specificity (96.80% [90.12–99.01]) than limb tools (89.69% [84.02–93.51]).

Heterogeneity: Significant heterogeneity (I^2^ > 75%) was observed across ‘wrists’. This variability likely reflects the diverse clinical settings in which commercial tools were tested, ranging from high-prevalence internal validation sets to low-prevalence real-world emergency department cohorts.

### Meta-analyses: researcher-developed models

**Fig. 3:**
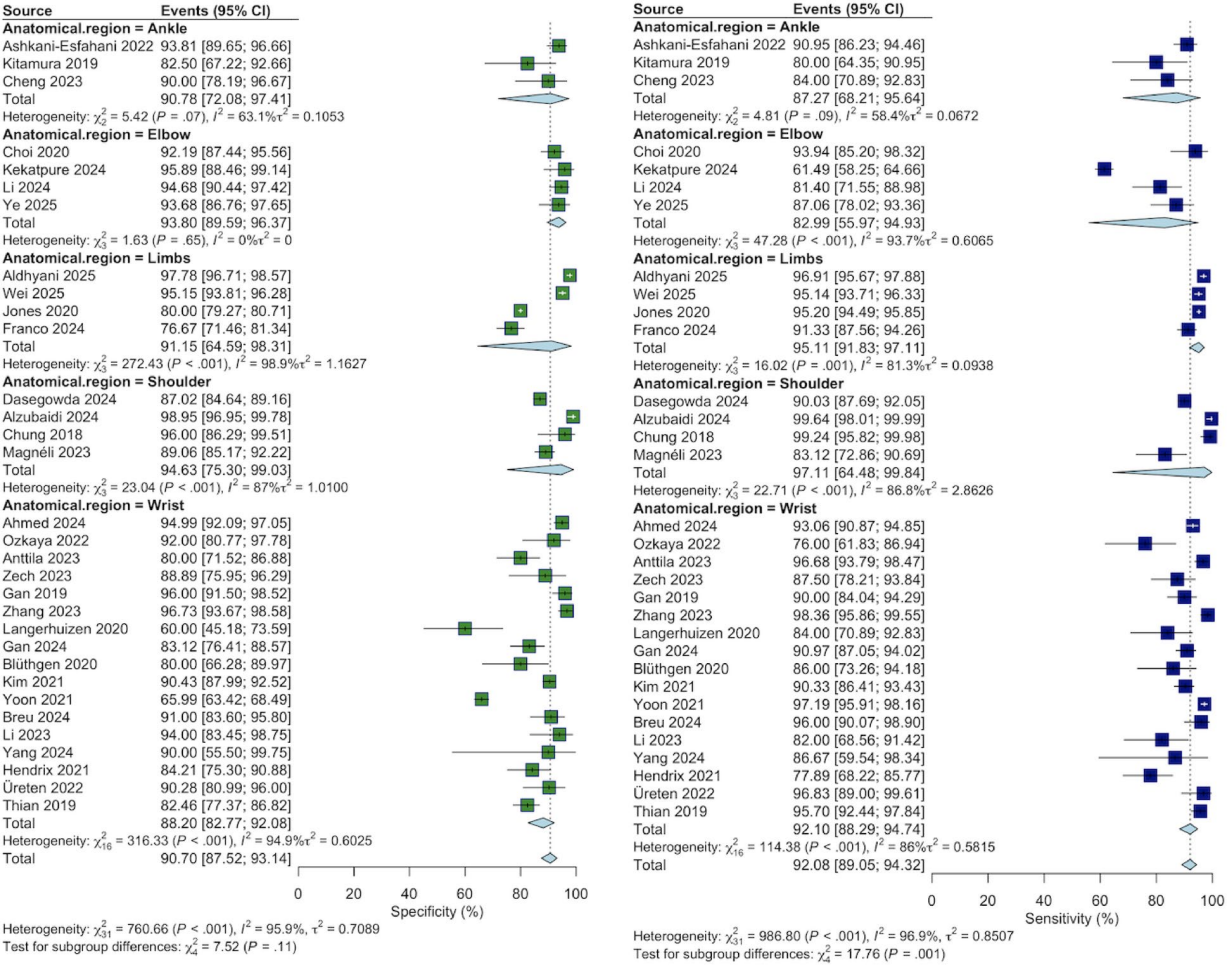
Forest plots of the diagnostic accuracy of researcher-developed models stratified by anatomical region

A random-effects meta-analysis was performed on 32 researcher-developed models (Fig. 3). Three studies were excluded from the quantitative synthesis because of a lack of reported contingency table data (true positives, false negatives, true negatives, and false positives) required for pooling [48, 52, 55]. Using a random-effects model was not feasible for four anatomical subgroups because too few studies were available: foot [28, 57], hand [36], and upper limb [61].

Sensitivity: The overall pooled sensitivity was 92.08% (95% CI 89.05–94.32) with very high heterogeneity (I^2^= 96.9%). The pooled subgroup sensitivities were as follows: ankle 87.27% (68.21–95.64), elbow 82.99% (55.97–94.93), limbs 95.11% (91.83–97.11), shoulder 97.11% (64.48–99.84), and wrist 92.10% (88.29–94.74).

Specificity: The overall pooled specificity was 90.70% (95% CI 87.52–93.14) with very high heterogeneity (I^2^= 95.9%). The pooled subgroup specificities were as follows: ankle 90.78% (72.08–97.41), elbow 93.80% (89.59–96.37), limbs 91.15% (64.59–98.31), shoulder 94.63% (75.30–99.03), and wrist 88.20% (82.77–92.08).

### Comparison between researcher-developed and commercial models

Notably, the pooled specificity of commercial limb tools (89.69%; CI 84.02–93.51) was lower than that of the best-performing researcher-developed models (e.g., shoulder specificity of 94.63%; CI 75.30–99.03). This pattern may reflect “spectrum bias,” as commercial tools are more frequently evaluated in consecutive clinical workflows where the prevalence of normal radiographs is higher, thereby challenging the algorithm’s ability to minimize false positives.

### Risk of bias assessment

The QUADAS-2 framework was used to assess the risk of bias, as shown in the table in the appendix. The major challenge was patient selection: many datasets were case-enriched/curated or narrowly scoped (e.g., pediatric-only or single-region cohorts), limiting representativeness for routine VFC case mixes. In contrast, studies using consecutive ED radiographs [33, 50] or real-world evaluations of commercial tools [16] have shown a lower selection risk. Flow & timing was generally low risk because index and reference assessments occurred within the same clinical episode.

The index-test domain was not uniformly low. The risk was lower when a locked commercial version was tested at a prespecified operating point with clear separation from training/tuning data and higher concerns in prototype studies using internal-only splits or tuned thresholds [54]. The reference-standard risk was lowest with independent multireader consensus and/or objective confirmation (e.g., CT/MRI) [18] and higher when truth relied on a single clinical report or nonclinical dataset labels [21]. Applicability mirrored these patterns, which are generally acceptable for index tests in commercial products, but greater concerns for selection (pediatric/single-region studies) and for nonclinical label sets. For NLP, applicability is language dependent; one study used software trained to recognize Dutch radiology reports [66].

## Discussion

### Current landscape of AI tools for virtual fracture clinics

Our systematic review identified established AI tools for fracture detection, dominated by image-analysis systems with emerging interest in text-based decision support [9]. Other pathologies, such as dislocation and joint effusion, can also be identified. Multiple technical approaches may be used to successfully identify fractures and other pathologies on plain radiographs, although translation to clinical practice requires addressing implementation challenges beyond diagnostic accuracy alone. The performance of fracture detection tools reported across studies was encouraging.

### Understanding how these systems work

Contemporary fracture detection AI employs deep learning architectures, primarily CNNs, that learn hierarchical visual features from large radiograph datasets. Unlike traditional computer-aided detection, which relies on handcrafted features, these systems automatically discover discriminative patterns through supervised learning on expert-annotated images [68].

The development of commercial tools identified in our review typically followed a multistage process. First, radiographs undergo preprocessing (normalization, augmentation) before passing through deep neural networks with millions of learnable parameters. These networks extract increasingly abstract features through successive convolutional layer edges and textures in early layers, anatomical structures in middle layers, and fracture-specific patterns in deeper layers. The final layers generate outputs tailored to clinical needs: probability scores for triage, bounding boxes for localization, or heatmaps highlighting regions of concern [68].

Different architectural choices reflect different clinical priorities. Region-based networks (R-CNN variants) excel at precise localization but require more computational resources. Lightweight architectures such as YOLO enable real-time processing suitable for high-volume emergency departments. Ensemble methods that combine multiple models can improve robustness but increase complexity and deployment costs [69].

### Diagnostic performance and quantitative synthesis

AI for fracture detection has matured into a high-performance diagnostic aid, with pooled sensitivities exceeding 92% for both commercial and researcher-developed models. Wrist-specific commercial tools achieved a pooled specificity (96.8% [90.12–99.01]) compared with multiregional “limb” tools (89.69% [84.02–93.51]). These findings suggest that anatomical targeting may effectively reduce false-positive rates.

### Implementation pathways and regulatory context

The NICE EVA provides a structured pathway for implementing AI fracture detection in NHS settings. The conditional recommendations for BoneView, RBfracture, Rayvolve, TechCare Alert, and qMSK establish these tools as decision aids rather than autonomous systems, requiring clear governance frameworks and ongoing evidence generation [4].

Several implementation models exist. The most conservative approach positions AI as a “second reader” that flags cases for human review without influencing initial interpretation. More integrated models use AI for worklist prioritization, ensuring that suspected fractures receive expedited specialist review [70]. The most advanced implementations explore AI-assisted triage, where algorithms help route patients to appropriate care pathways, although these require robust safety nets and exception-handling protocols [4, 71].

Technical integration presents practical challenges. PACS compatibility, security, data governance, and workflow disruption must be carefully managed. Successful implementations may begin with pilot phases in controlled settings before broader deployment. Careful performance review and the design of safeguards are required for implementation. “Silent mode” validation, where AI runs parallel to standard care without influencing decisions, helps build institutional confidence and evidence of local performance [4, 71].

#### Clinical context integration

Our review highlights a gap in the ability to combine imaging with clinical documentation. Virtual fracture clinics make decisions on the basis of both radiographs and emergency department notes describing the mechanism of injury, examination findings, and functional status [72]. Current AI tools address only half this equation [6, 73].

Further studies focused on multimodal systems are needed. Multimodal systems might employ late fusion architectures [74] where separate image and text models generate independent predictions before being combined. Alternatively, early fusion approaches could learn joint assessments of images and text, potentially discovering novel image-text correlations relevant to clinical outcomes. The optimal architecture likely depends on text data availability and quality, computational resources, and specific clinical objectives.

#### Limitations

While our review identified numerous AI X-ray assessment tools, external validation remains uncommon, with most studies evaluating models on data from the same institutions where they were developed [75]. Although radiograph-based AI shows high performance for fracture detection, its performance for other injuries commonly reviewed via VFC pathways (e.g., reduced dislocations/subluxations and significant soft-tissue injuries) remains largely untested in the current evidence base. Workflow and economic outcomes receive minimal attention [5]. The predominance of retrospective designs limits the understanding of prospective clinical impact [9]. Additionally, few studies have reported calibration metrics, confidence intervals, or failure mode analyses that can inform safe deployment [76–78].

Studies were restricted to papers published in English, which may lead to the underreporting of potential tools. The expansion of AI tools has been rapid; this review did not capture newer tools where outcomes have not yet been published in the medical literature.

Although our risk of bias assessment (Supplementary Tables 1–3) revealed high diagnostic accuracy across many studies, many researcher-developed models have been validated on “case-enriched” datasets where fractures and normal radiographs are balanced (e.g., a 50:50 split). In a live clinical setting with low fracture incidence, even a highly specific tool can generate a substantial number of false positives. However, in a VFC setting, where referrals are only made for confirmed or suspected fractures, this is less of a concern than AI detection tools used for the first review in the emergency department.

### The multimodal gap

Current tools almost exclusively address imaging. We identified only two studies exploring NLP for fracture-related text, and no commercial tools currently integrate clinical notes with image analysis. Future development must focus on multimodal AI architectures (e.g., late fusion systems) that can process both pixel and text data to support holistic triage decisions.

Most datasets are weighted toward adult populations. There is a risk that general tools may underperform in subgroups, such as pediatric patients with developing ossification centers or elderly patients with osteopenia, unless specifically calibrated for them. To mitigate “automation bias”, where clinicians overrely on AI outputs, implementation must include local calibration. A tool validated in a major trauma center may not perform identically in a district general hospital due to demographic differences. Implementation Strategy: We recommend that institutions adhere to the NICE Early Value Assessment recommendations by establishing clear governance for AI-clinician disagreements.

#### Recommendations for future research

Future studies may use DECIDE-AI and CLAIM checklists to ensure the transparent reporting of reference standards and training demographics [78]. Research should focus on establishing acceptable error rates for systems that combine imaging and text. Hospitals may conduct prospective phases where AI runs in the background without influencing care. This establishes baseline error rates and ‘failure modes’ safely before full deployment.

## Conclusion

AI VFCs could revolutionize acute orthopedic care, with current imaging tools showing encouraging performance. Clinical adoption is currently limited by a lack of multimodal (image + text) integration. To ensure safe deployment, hospitals may move beyond ‘plug-and-play’ adoption, prioritizing local calibration, ‘silent mode’ testing, and the development of equitable algorithms that perform robustly across diverse patient demographics.

## Supplementary Information

Below is the link to the electronic supplementary material.

Supplementary Material 1

## Data Availability

The datasets used and/or analyzed during the current study are available from the corresponding author on reasonable request.

## Abbreviations

VFC: Virtual fracture clinic
AI: Artificial intelligence
DL: Deep learning
NLP: Natural language processing
ED: Emergency Department
PACS: Picture archiving and communication systems
CNN: Convolutional neural networks
QUADAS: Quality assessment of diagnostic accuracy studies
NICE: National Institute for Health and Care Excellence
EVA: Early value assessment

## References

[1] Vardy J, Jenkins PJ, Clark K, et al. Effect of a redesigned fracture management pathway and ‘virtual’ fracture clinic on ED performance. BMJ Open. 2014;4(6):e005282. 24928593 10.1136/bmjopen-2014-005282 PMC4067811

[2] McKirdy A, Imbuldeniya AM. The clinical and cost effectiveness of a virtual fracture clinic service: an interrupted time series analysis and before-and-after comparison. Bone Joint Res. 2017;6(5):259–69. 28473333 10.1302/2046-3758.65.BJR-2017-0330.R1 PMC5457647

[3] Khan SA, Asokan A, Handford C, Logan P, Moores T. How useful are virtual fracture clinics? Bone Joint Open. 2020;1(11):683–90. 33263108 10.1302/2633-1462.111.BJO-2020-0107.R1 PMC7690759

[4] National Institute for Health and Care Excellence (NICE). Artificial intelligence (AI) technologies to help detect fractures on X-rays in urgent care: early value assessment (HTE20) [Internet]. London: NICE. 2025 Jan 14 [cited 2025 Sep 12]. Available from: https://www.nice.org.uk/guidance/hte20

[5] Kuo RYL, Harrison C, Curran TA, et al. Artificial intelligence in fracture detection: a systematic review and meta-analysis. Radiology. 2022;304(1):50–62. 35348381 10.1148/radiol.211785 PMC9270679

[6] Stewart J, Lu J, Goudie A, et al. Applications of natural Language processing at emergency department triage: a narrative review. PLoS ONE. 2023;18(12):e0279953. 38096321 10.1371/journal.pone.0279953 PMC10721204

[7] Porto BM. Improving triage performance in emergency departments using machine learning and natural Language processing: a systematic review. BMC Emerg Med. 2024;24(1):219. 39558255 10.1186/s12873-024-01135-2 PMC11575054

[8] Sterling NW, Patzer RE, Di M, Schrager JD. Prediction of emergency department patient disposition based on natural Language processing of triage notes. Int J Med Inf. 2019;129:184–8. 10.1016/j.ijmedinf.2019.06.008 31445253

[9] Nowroozi A, Salehi MA, Shobeiri P, et al. Artificial intelligence diagnostic accuracy in fracture detection from plain radiographs and comparing it with clinicians: a systematic review and meta-analysis. Clin Radiol. 2024;79(8):579–88. 38772766 10.1016/j.crad.2024.04.009

[10] Jung J, Dai J, Liu B, Wu Q. Artificial intelligence in fracture detection with different image modalities and data types: a systematic review and meta-analysis. PLOS Digit Health. 2024;3(1):e0000438. 38289965 10.1371/journal.pdig.0000438 PMC10826962

[11] Suen K, Zhang R, Kutaiba N. Accuracy of wrist fracture detection on radiographs by artificial intelligence compared to human clinicians: a systematic review and meta-analysis. Eur J Radiol. 2024;178:111593. 38981178 10.1016/j.ejrad.2024.111593

[12] IntHout J, Ioannidis JP, Borm GF. The Hartung-Knapp-Sidik-Jonkman method for random effects meta-analysis is straightforward and considerably outperforms the standard DerSimonian-Laird method. BMC Med Res Methodol. 2014;14:25. 24548571 10.1186/1471-2288-14-25 PMC4015721

[13] Whiting PF, Rutjes AW, Westwood ME, et al. QUADAS-2: a revised tool for the quality assessment of diagnostic accuracy studies. Ann Intern Med. 2011;155(8):529–36. 22007046 10.7326/0003-4819-155-8-201110180-00009

[14] Hayashi D, Kompel AJ, Ventre J, et al. Automated detection of acute appendicular skeletal fractures in pediatric patients using deep learning. Skeletal Radiol. 2022;51(11):2129–39. 35522332 10.1007/s00256-022-04070-0

[15] Duron L, Ducarouge A, Gillibert A, et al. Assessment of an AI aid in detection of adult appendicular skeletal fractures by emergency physicians and radiologists: a multicenter cross-sectional diagnostic study. Radiology. 2021;300(1):120–9. 33944629 10.1148/radiol.2021203886

[16] Bousson V, Attané G, Benoist N, et al. Artificial intelligence for detecting acute fractures in patients admitted to an emergency department: real-life performance of three commercial algorithms. Acad Radiol. 2023;30(10):2118–39. 37468377 10.1016/j.acra.2023.06.016

[17] Guermazi A, Tannoury C, Kompel AJ, et al. Improving radiographic fracture recognition performance and efficiency using artificial intelligence. Radiology. 2022;302(3):627–36. 34931859 10.1148/radiol.210937

[18] Dell’Aria A, Tack D, Saddiki N, et al. Radiographic detection of post-traumatic bone fractures: contribution of artificial intelligence software to the analysis of senior and junior radiologists. J Belg Soc Radiol. 2024;108(1):44. 38680721 10.5334/jbsr.3574 PMC11049681

[19] Cohen M, Puntonet J, Sanchez J, et al. Artificial intelligence vs. radiologist: accuracy of wrist fracture detection on radiographs. Eur Radiol. 2023;33(6):3974–83. 36515712 10.1007/s00330-022-09349-3

[20] Russe MF, Rebmann P, Tran PH, et al. AI-based X-ray fracture analysis of the distal radius: accuracy between representative classification, detection and segmentation deep learning models for clinical practice. BMJ Open. 2024;14(1):e076954. 38262641 10.1136/bmjopen-2023-076954 PMC10823998

[21] Ramadanov N, John P, Hable R, et al. Artificial intelligence-guided distal radius fracture detection on plain radiographs in comparison with human raters. J Orthop Surg Res. 2025;20(1):468. 40380226 10.1186/s13018-025-05888-9 PMC12083173

[22] Bachmann R, Gunes G, Hangaard S, et al. Improving traumatic fracture detection on radiographs with artificial intelligence support: a multi-reader study. BJR Open. 2024;6(1):tzae011. 38757067 10.1093/bjro/tzae011 PMC11096271

[23] Ziegner M, Pape J, Lacher M, et al. Real-life benefit of artificial intelligence-based fracture detection in a pediatric emergency department. Eur Radiol. 2025;35(10):5881–90. 40192806 10.1007/s00330-025-11554-9 PMC12417293

[24] Dupuis M, Delbos L, Veil R, Adamsbaum C. External validation of a commercially available deep learning algorithm for fracture detection in children. Diagn Interv Imaging. 2022;103(3):151–9. 34810137 10.1016/j.diii.2021.10.007

[25] Díaz Moreno A, Cano Alonso R, Fernández Alfonso A et al. Diagnostic performance of an artificial intelligence software for the evaluation of bone X-Ray examinations referred from the emergency department. Diagnostics (Basel). 2025;15(4). 10.3390/diagnostics15040491 PMC11854177 40002642

[26] Lee KC, Choi IC, Kang CH et al. Clinical validation of an artificial intelligence model for detecting distal Radius, ulnar Styloid, and scaphoid fractures on conventional wrist radiographs. Diagnostics (Basel). 2023;13(9). 10.3390/diagnostics13091657 PMC10178713 37175048

[27] Ye Q, Wang Z, Lou Y, et al. Deep learning approach based on a patch residual for pediatric Supracondylar subtle fracture detection. Biomol Biomed. 2025;25(7):1631–46. 39829118 10.17305/bb.2024.11341 PMC12097401

[28] Kim T, Goh TS, Lee JS, Lee JH, Kim H, Jung ID. Transfer learning-based ensemble convolutional neural network for accelerated diagnosis of foot fractures. Phys Eng Sci Med. 2023;46(1):265–77. 36625995 10.1007/s13246-023-01215-w

[29] Alzubaidi L, Salhi A, M AF, et al. Trustworthy deep learning framework for the detection of abnormalities in X-ray shoulder images. PLoS ONE. 2024;19(3):e0299545. 38466693 10.1371/journal.pone.0299545 PMC10927121

[30] Magnéli M, Ling P, Gislén J, et al. Deep learning classification of shoulder fractures on plain radiographs of the humerus, scapula and clavicle. PLoS ONE. 2023;18(8):e0289808. 37647274 10.1371/journal.pone.0289808 PMC10468075

[31] Breu R, Avelar C, Bertalan Z, et al. Artificial intelligence in traumatology. Bone Joint Res. 2024;13(10):588–95. 39417424 10.1302/2046-3758.1310.BJR-2023-0275.R3 PMC11484119

[32] Yang TH, Sun YN, Li RS, Horng MH. The detection and classification of scaphoid fractures in radiograph by using a convolutional neural network. Diagnostics (Basel). 2024;14:21. 10.3390/diagnostics14212425 PMC11545356 39518391

[33] Anttila TT, Karjalainen TV, Mäkelä TO, et al. Detecting distal radius fractures using a segmentation-based deep learning model. J Digit Imaging. 2023;36(2):679–87. 36542269 10.1007/s10278-022-00741-5 PMC10039188

[34] Langerhuizen DWG, Bulstra AEJ, Janssen SJ, et al. Is deep learning on par with human observers for detection of radiographically visible and occult fractures of the scaphoid? Clin Orthop Relat Res. 2020;478(11):2653–9. 32452927 10.1097/CORR.0000000000001318 PMC7571968

[35] Kitamura G, Chung CY, Moore BE. 2nd. Ankle fracture detection utilizing a convolutional neural network ensemble implemented with a small Sample, de Novo Training, and multiview incorporation. J Digit Imaging. 2019;32(4):672–7. 31001713 10.1007/s10278-018-0167-7 PMC6646476

[36] Axenhus M, Wallin A, Havela J, et al. Automated diagnosis and classification of metacarpal and phalangeal fractures using a convolutional neural network: a retrospective data analysis study. Acta Orthop. 2025;96:13–8. 39786203 10.2340/17453674.2024.42702 PMC11714779

[37] Choi JW, Cho YJ, Lee S, et al. Using a Dual-Input convolutional neural network for automated detection of pediatric Supracondylar fracture on conventional radiography. Invest Radiol. 2020;55(2):101–10. 31725064 10.1097/RLI.0000000000000615

[38] Chung SW, Han SS, Lee JW, et al. Automated detection and classification of the proximal humerus fracture by using deep learning algorithm. Acta Orthop. 2018;89(4):468–73. 29577791 10.1080/17453674.2018.1453714 PMC6066766

[39] Kekatpure A, Kekatpure A, Deshpande S, Srivastava S. Development of a diagnostic support system for distal humerus fracture using artificial intelligence. Int Orthop. 2024;48(5):1303–11. 38499714 10.1007/s00264-024-06125-4

[40] Ozkaya E, Topal FE, Bulut T, Gursoy M, Ozuysal M, Karakaya Z. Evaluation of an artificial intelligence system for diagnosing scaphoid fracture on direct radiography. Eur J Trauma Emerg Surg. 2022;48(1):585–92. 32862314 10.1007/s00068-020-01468-0

[41] Hendrix N, Scholten E, Vernhout B, et al. Development and validation of a convolutional neural network for automated detection of scaphoid fractures on conventional radiographs. Radiol Artif Intell. 2021;3(4):e200260. 34350413 10.1148/ryai.2021200260 PMC8329964

[42] Kim MW, Jung J, Park SJ, et al. Application of convolutional neural networks for distal radio-ulnar fracture detection on plain radiographs in the emergency room. Clin Exp Emerg Med. 2021;8(2):120–7. 34237817 10.15441/ceem.20.091 PMC8273672

[43] Li J, Hu W, Wu H, et al. Detection of hidden pediatric elbow fractures in X-ray images based on deep learning. J Radiation Res Appl Sci. 2024;17(2):100893.

[44] Gan K, Liu Y, Zhang T, et al. Deep learning model for automatic identification and classification of distal radius fracture. J Imaging Inf Med. 2024;37(6):2874–82. 10.1007/s10278-024-01144-4 PMC11612100 38862852

[45] Dasegowda G, Sato JY, Elton DC, et al. No code machine learning: validating the approach on use-case for classifying clavicle fractures. Clin Imaging. 2024;112:110207. 38838448 10.1016/j.clinimag.2024.110207

[46] Aldhyani T, Ahmed ZAT, Alsharbi BM, et al. Diagnosis and detection of bone fracture in radiographic images using deep learning approaches. Front Med (Lausanne). 2024;11:1506686. 39927268 10.3389/fmed.2024.1506686 PMC11803505

[47] Wei W, Huang Y, Zheng J, et al. YOLOv11-based multi-task learning for enhanced bone fracture detection and classification in X-ray images. J Radiation Res Appl Sci. 2025;18(1):101309.

[48] Binh LN, Nhu NT, Vy VPT, et al. Multi-Class deep learning model for detecting pediatric distal forearm fractures based on the AO/OTA classification. J Imaging Inf Med. 2024;37(2):725–33. 10.1007/s10278-024-00968-4 PMC11031555 38308069

[49] Ahmed A, Imran AS, Manaf A, Kastrati Z, Daudpota SM. Enhancing wrist abnormality detection with YOLO: analysis of state-of-the-art single-stage detection models. Biomed Signal Process Control. 2024;93:106144.

[50] Thian YL, Li Y, Jagmohan P, Sia D, Chan VEY, Tan RT. Convolutional neural networks for automated fracture detection and localization on wrist radiographs. Radiol Artif Intell. 2019;1(1):e180001. 33937780 10.1148/ryai.2019180001 PMC8017412

[51] Ashkani-Esfahani S, Mojahed Yazdi R, Bhimani R, et al. Detection of ankle fractures using deep learning algorithms. Foot Ankle Surg. 2022;28(8):1259–65. 35659710 10.1016/j.fas.2022.05.005

[52] Kyung S, Jang M, Park S, Yoon HM, Hong GS, Kim N. Supervised representation learning based on various levels of pediatric radiographic views for transfer learning. Sci Rep. 2024;14(1):7551. 38555414 10.1038/s41598-024-58163-y PMC10981659

[53] Cheng CT, Hsu CP, Ooyang CH, et al. Evaluation of ensemble strategy on the development of multiple view ankle fracture detection algorithm. Br J Radiol. 2023;96(1145):20220924. 36930721 10.1259/bjr.20220924 PMC10161902

[54] Yoon AP, Lee YL, Kane RL, Kuo CF, Lin C, Chung KC. Development and validation of a deep learning model using convolutional neural networks to identify scaphoid fractures in radiographs. JAMA Netw Open. 2021;4(5):e216096. 33956133 10.1001/jamanetworkopen.2021.6096 PMC8103226

[55] Rashid T, Zia MS, Najam Ur R, Meraj T, Rauf HT, Kadry S. A minority class balanced approach using the DCNN-LSTM method to detect human wrist fracture. Life (Basel). 2023;13(1). 10.3390/life13010133 PMC9861673 36676082

[56] Jones RM, Sharma A, Hotchkiss R, et al. Assessment of a deep-learning system for fracture detection in musculoskeletal radiographs. NPJ Digit Med. 2020;3:144. 33145440 10.1038/s41746-020-00352-w PMC7599208

[57] Yu Q, Liu Y, Li H, et al. Multi-task learning for calcaneus fracture diagnosis of X-ray images. Biomed Signal Process Control. 2025;99:106843.

[58] Üreten K, Sevinç HF, İğdeli U, Onay A, Maraş Y. Use of deep learning methods for hand fracture detection from plain hand radiographs. Ulus Travma Acil Cerrahi Derg. 2022;28(2):196–201. 35099027 10.14744/tjtes.2020.06944 PMC10443147

[59] Blüthgen C, Becker AS, Vittoria de Martini I, Meier A, Martini K, Frauenfelder T. Detection and localization of distal radius fractures: deep learning system versus radiologists. Eur J Radiol. 2020;126:108925. 32193036 10.1016/j.ejrad.2020.108925

[60] Franco PN, Maino C, Mariani I, et al. Diagnostic performance of an AI algorithm for the detection of appendicular bone fractures in pediatric patients. Eur J Radiol. 2024;178:111637. 39053306 10.1016/j.ejrad.2024.111637

[61] Zech JR, Ezuma CO, Patel S, et al. Artificial intelligence improves resident detection of pediatric and young adult upper extremity fractures. Skeletal Radiol. 2024;53(12):2643–51. 38695875 10.1007/s00256-024-04698-0

[62] Zech JR, Carotenuto G, Igbinoba Z, et al. Detecting pediatric wrist fractures using deep-learning-based object detection. Pediatr Radiol. 2023;53(6):1125–34. 36650360 10.1007/s00247-023-05588-8

[63] Gan K, Xu D, Lin Y, et al. Artificial intelligence detection of distal radius fractures: a comparison between the convolutional neural network and professional assessments. Acta Orthop. 2019;90(4):394–400. 30942136 10.1080/17453674.2019.1600125 PMC6718190

[64] Zhang J, Li Z, Lin H, et al. Deep learning assisted diagnosis system: improving the diagnostic accuracy of distal radius fractures. Front Med (Lausanne). 2023;10:1224489. 37663656 10.3389/fmed.2023.1224489 PMC10471443

[65] Li T, Yin Y, Yi Z, Guo Z, Guo Z, Chen S. Evaluation of a convolutional neural network to identify scaphoid fractures on radiographs. J Hand Surg Eur Vol. 2023;48(5):445–50. 36205038 10.1177/17531934221127092

[66] Olthof AW, Shouche P, Fennema EM, et al. Machine learning based natural Language processing of radiology reports in orthopaedic trauma. Comput Methods Programs Biomed. 2021;208:106304. 34333208 10.1016/j.cmpb.2021.106304

[67] Zech JR, Jaramillo D, Altosaar J, Popkin CA, Wong TT. Artificial intelligence to identify fractures on pediatric and young adult upper extremity radiographs. Pediatr Radiol. 2023;53(12):2386–97. 37740031 10.1007/s00247-023-05754-y

[68] Litjens G, Kooi T, Bejnordi BE, et al. A survey on deep learning in medical image analysis. Med Image Anal. 2017;42:60–88. 28778026 10.1016/j.media.2017.07.005

[69] Muhammad D, Bendechache M. Unveiling the black box: a systematic review of explainable artificial intelligence in medical image analysis. Comput Struct Biotechnol J. 2024;24:542–60. 39252818 10.1016/j.csbj.2024.08.005 PMC11382209

[70] The Royal College of Radiologists. Clinical Radiology: AI deployment fundamentals for medical imaging [Internet]. London (UK): The Royal College of Radiologists; 2024 Nov [cited 2025 Sep 12]. Available from: https://www.rcr.ac.uk/media/sbdhwnfl/ai-deployment-fundamentals-for-medical-imaging-2024.pdf

[71] Joshi I, Cushnan D. A buyer’s guide to AI in health and care: 10 questions for making well-informed procurement decisions about products that use AI [Internet]. London: NHSX; 2020 Nov [cited 2025 Sep 12]. Available from: https://webarchive.nationalarchives.gov.uk/ukgwa/20230504130145mp_/https%3A//transform.england.nhs.uk/media/documents/NHSX_A_Buyers_Guide_to_AI_in_Health_and_Care.pdf

[72] Da’Costa A, Teke J, Origbo JE, Osonuga A, Egbon E, Olawade DB. AI-driven triage in emergency departments: a review of benefits, challenges, and future directions. Int J Med Inf. 2025;197:105838. 10.1016/j.ijmedinf.2025.105838 39965433

[73] Wang Y, Mehrabi S, Sohn S, Atkinson EJ, Amin S, Liu H. Natural Language processing of radiology reports for identification of skeletal site-specific fractures. BMC Med Inf Decis Mak. 2019;19(Suppl 3):73. 10.1186/s12911-019-0780-5 PMC6448178 30943952

[74] Huang SC, Pareek A, Seyyedi S, Banerjee I, Lungren MP. Fusion of medical imaging and electronic health records using deep learning: a systematic review and implementation guidelines. NPJ Digit Med. 2020;3:136. 33083571 10.1038/s41746-020-00341-z PMC7567861

[75] Yu AC, Mohajer B, Eng J. External validation of deep learning algorithms for radiologic diagnosis: a systematic review. Radiol Artif Intell. 2022;4(3):e210064. 35652114 10.1148/ryai.210064 PMC9152694

[76] Vasey B, Nagendran M, Campbell B, et al. Reporting guideline for the early stage clinical evaluation of decision support systems driven by artificial intelligence: DECIDE-AI. BMJ. 2022;377:e070904. 35584845 10.1136/bmj-2022-070904 PMC9116198

[77] Liu X, Rivera SC, Moher D, Calvert MJ, Denniston AK. Reporting guidelines for clinical trial reports for interventions involving artificial intelligence: the CONSORT-AI extension. BMJ. 2020;370:m3164. 32909959 10.1136/bmj.m3164 PMC7490784

[78] Mongan J, Moy L, Kahn CE. Jr. Checklist for artificial intelligence in medical imaging (CLAIM): a guide for authors and reviewers. Radiol Artif Intell. 2020;2(2):e200029. 33937821 10.1148/ryai.2020200029 PMC8017414

