# Supplementary material for: Artificial intelligence in virtual fracture clinics: a systematic review of imaging and clinical-text tools
Source: J Orthop Surg Res. 2026 Feb 6;21:176. doi: 10.1186/s13018-025-06656-5 (PMC12973765; doi:10.1186/s13018-025-06656-5)
Supplement: Supplementary file 1 — Supplementary Material 1 [file 13018_2025_6656_MOESM1_ESM.docx]

| **Section and Topic** | **Item #** | **Checklist item** | **Location where item**  **is reported** |
| --- | --- | --- | --- |
| **TITLE** | | |  |
| Title | 1 | Identify the report as a systematic review. | 1 |
| **ABSTRACT** | | |  |
| Abstract | 2 | See the PRISMA 2020 for Abstracts checklist. | 1 |
| **INTRODUCTION** | | |  |
| Rationale | 3 | Describe the rationale for the review in the context of existing knowledge. | 3-4 |
| Objectives | 4 | Provide an explicit statement of the objective(s) or question(s) the review addresses. | 3-4 |
| **METHODS** | | |  |
| Eligibility criteria | 5 | Specify the inclusion and exclusion criteria for the review and how studies were grouped for the syntheses. | 4-5 |
| Information sources | 6 | Specify all databases, registers, websites, organisations, reference lists and other sources searched or consulted to identify studies. Specify the date when each source was last searched or consulted. | 4-5 |
| Search strategy | 7 | Present the full search strategies for all databases, registers and websites, including any filters and limits used. | Appendix |
| Selection process | 8 | Specify the methods used to decide whether a study met the inclusion criteria of the review, including how many reviewers screened each record and each report retrieved, whether they worked independently, and if applicable, details of automation tools used in the process. | 4-5 |
| Data collection process | 9 | Specify the methods used to collect data from reports, including how many reviewers collected data from each report, whether they worked independently, any processes for obtaining or confirming data from study investigators, and if applicable, details of automation tools used in the process. | 4-5 |
| Data items | 10a | List and define all outcomes for which data were sought. Specify whether all results that were compatible with each outcome domain in each study were sought (e.g. for all measures, time points, analyses), and if not, the methods used to decide which results to collect. | 4-5 |
|  | 10b | List and define all other variables for which data were sought (e.g. participant and intervention characteristics, funding sources). Describe any assumptions made about any missing or unclear information. | 4-5 |
| Study risk of bias assessment | 11 | Specify the methods used to assess risk of bias in the included studies, including details of the tool(s) used, how many reviewers assessed each study and whether they worked independently, and if applicable, details of automation tools used in the process. | Appendix |
| Effect measures | 12 | Specify for each outcome the effect measure(s) (e.g. risk ratio, mean difference) used in the synthesis or presentation of results. | 4-5 |
| Synthesis methods | 13a | Describe the processes used to decide which studies were eligible for each synthesis (e.g. tabulating the study intervention characteristics and comparing against the planned groups for each synthesis (item #5)). | 4-5 |
|  | 13b | Describe any methods required to prepare the data for presentation or synthesis, such as handling of missing summary statistics, or data conversions. | 4-5 |
|  | 13c | Describe any methods used to tabulate or visually display results of individual studies and syntheses. | 4-5 |
|  | 13d | Describe any methods used to synthesize results and provide a rationale for the choice(s). If meta-analysis was performed, describe the model(s), method(s) to identify the presence and extent of statistical heterogeneity, and software package(s) used. | 5 |
|  | 13e | Describe any methods used to explore possible causes of heterogeneity among study results (e.g. subgroup analysis, meta-regression). | 5 |
|  | 13f | Describe any sensitivity analyses conducted to assess robustness of the synthesized results. | No |
| Reporting bias assessment | 14 | Describe any methods used to assess risk of bias due to missing results in a synthesis (arising from reporting biases). | No |
| Certainty assessment | 15 | Describe any methods used to assess certainty (or confidence) in the body of evidence for an outcome. | No |

**
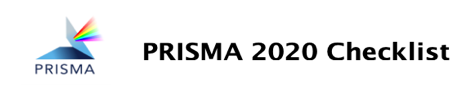
Supplement I. PRISMA 2020 Checklist**

| **Section and Topic** | **Item #** | **Checklist item** | **Location where item**  **is reported** |
| --- | --- | --- | --- |
| **RESULTS** | | |  |
| Study selection | 16a | Describe the results of the search and selection process, from the number of records identified in the search to the number of studies included in the review, ideally using a flow diagram. | 6 |
|  | 16b | Cite studies that might appear to meet the inclusion criteria, but which were excluded, and explain why they were excluded. | No |
| Study characteristics | 17 | Cite each included study and present its characteristics. | 6-11 |
| Risk of bias in studies | 18 | Present assessments of risk of bias for each included study. | Appendix |
| Results of individual studies | 19 | For all outcomes, present, for each study: (a) summary statistics for each group (where appropriate) and (b) an effect estimate and its precision (e.g. confidence/credible interval), ideally using structured tables or plots. | 8-14 |
| Results of syntheses | 20a | For each synthesis, briefly summarise the characteristics and risk of bias among contributing studies. | 8-14 |
|  | 20b | Present results of all statistical syntheses conducted. If meta-analysis was done, present for each the summary estimate and its precision (e.g. confidence/credible interval) and measures of statistical heterogeneity. If comparing groups, describe the direction of the effect. | 11-13 |
|  | 20c | Present results of all investigations of possible causes of heterogeneity among study results. | 11-13 |
|  | 20d | Present results of all sensitivity analyses conducted to assess the robustness of the synthesized results. | No |
| Reporting biases | 21 | Present assessments of risk of bias due to missing results (arising from reporting biases) for each synthesis assessed. | No |
| Certainty of evidence | 22 | Present assessments of certainty (or confidence) in the body of evidence for each outcome assessed. | No |
| **DISCUSSION** | | |  |
| Discussion | 23a | Provide a general interpretation of the results in the context of other evidence. | 15-18 |
|  | 23b | Discuss any limitations of the evidence included in the review. | 17-18 |
|  | 23c | Discuss any limitations of the review processes used. | 17-19 |
|  | 23d | Discuss implications of the results for practice, policy, and future research. | 18-19 |
| **OTHER INFORMATION** | | |  |
| Registration and protocol | 24a | Provide registration information for the review, including register name and registration number, or state that the review was not registered. | 3 |
|  | 24b | Indicate where the review protocol can be accessed, or state that a protocol was not prepared. | 3 |
|  | 24c | Describe and explain any amendments to information provided at registration or in the protocol. | No |
| Support | 25 | Describe sources of financial or non-financial support for the review, and the role of the funders or sponsors in the review. | 25 |
| Competing interests | 26 | Declare any competing interests of review authors. | 25 |
| Availability of data, code and other materials | 27 | Report which of the following are publicly available and where they can be found: template data collection forms; data extracted from included studies; data used for all analyses; analytic code; any other materials used in the review. | 25 |

From: Page MJ, McKenzie JE, Bossuyt PM, Boutron I, Hoffmann TC, Mulrow CD, et al. The PRISMA 2020 statement: an updated guideline for reporting systematic reviews. BMJ 2021;372:n71. doi: 10.1136/bmj.n71. This work is licensed under CC BY 4.0. To view a copy of this license, visit https://creativecommons.org/licenses/by/4.0/

**Supplement II. Search Terms for Ovid**

1

exp Fractures/

2

(fractur* or bone break*).mp.

3

("osteoporotic fractur*" or "fragility fractur*" or "stress fractur*").mp.

4

(hip fractur* or femoral neck fractur* or intertrochanteric fractur* or subtrochanteric fractur*).mp.

5

(wrist fractur* or distal radius fractur* or scaphoid fractur*).mp.

6

(vertebral fractur* or spinal compression fractur*).mp.

7

(pelvic fractur* or acetabular fractur*).mp.

8

(ankle fractur* or tibial plafond fractur* or calcaneus fractur*).mp.

9

(humerus fractur* or proximal humerus fractur*).mp.

10

(tibia fractur* or tibial plateau fractur* or fibula fractur*).mp.

11

(clavicle fractur* or rib fractur*).mp.

12

(pathologic fractur* or greenstick fractur* or comminut* fractur*).mp.

13

(open fractur* or closed fractur*).mp.

14

("fracture clinic" or "fracture liaison" or nonunion or malunion).mp.

15

1 or 2 or 3 or 4 or 5 or 6 or 7 or 8 or 9 or 10 or 11 or 12 or 13 or 14

16

exp Artificial Intelligence/

17

exp Machine Learning/

18

exp Natural Language Processing/

19

(deep learning or transformer model or LLM or computer vision).mp.

20

("artificial intelligence" or "machine learning" or "deep learning" or NLP or "natural language processing").mp.

21

("neural network*" or connectionist model or "computational neural network").mp.

22

16 or 17 or 18 or 19 or 20 or 21

23

exp Triage/

24

triage.mp.

25

(schedul* or appointment*).mp.

26

("risk stratification" or "risk prediction").mp.

27

("decision support" or "clinical pathway" or "care pathway").mp.

28

23 or 24 or 25 or 26 or 27

29

exp Radiography/

30

(radiograph* or x-ray or xray or "plain film*").mp.

31

(digital radiograph* or "computed radiography" or "projection radiography").mp.

32

("image analys*" or "image process*" or CAD or "computer-aided diagnos*" or "computer-aided detection").mp.

33

(CNN or "convolutional neural network" or "feature extraction" or segmentation or "fracture detection" or "fracture localisation").mp.

34

29 or 30 or 31 or 32 or 33

35

15 and 22 and 28 and 34

**Supplement III. Signaling Questions Risk of Bias**

Signaling questions for the concerns about the Risk of Bias:

1. Patient selection

- Were patients enrolled consecutively or randomly, avoiding case–control designs and inappropriate exclusions?
- Was a case-control design avoided? (Did the study use a correct split sampling method and was the data evaluated on an external validation test set?)

2. Index test

- Were index-test results obtained/interpreted without knowledge of the reference standard?
- Was any decision threshold prespecified (i.e., not tuned on the test set)?
- Was the test set independent of training/tuning data (no patient overlaps or leakage)?
- (If reader-assist) Were readers blinded, and was the assist interface/workflow representative of clinical use?

3. Reference standard

- Is the reference standard likely to correctly classify fractures (e.g., ≥2-reader consensus or objective confirmation by CT/MRI/operative findings/follow-up)?
- Were reference-standard assessments independent of the index test (blinded)?

4. Flow and timing

- Was the interval between index test and reference standard appropriate?
- Did all participants receive the same reference standard?
- Were all enrolled patients included in the analysis, with indeterminate/uninterpretable results handled transparently?

Signaling questions for the concerns about applicability:

1. Patient selection

- Does the **setting and pathway** match VFC/urgent-care use (ED/minor injuries), with a **case mix** (age, anatomical regions, prevalence) relevant to the review question?
- Were **radiographic protocols/views** typical of urgent care?

2. Index test

- Is the AI product/version and intended use (reader-assist vs stand-alone) aligned with expected clinical deployment?
- Is the operating point/threshold and output presentation/workflow (e.g., PACS integration, triage flags) consistent with practice?

3. Reference standard

- Does the reference standard’s definition of “fracture” match the target condition (acute traumatic fracture on radiographs in the specified regions)?
- Is adjudication consistent with practice (multireader consensus or objective confirmation) for the same regions/time window?

**Supplement IV. QUADAS-2 risk of bias tables**

|  | Risk of Bias | | | | Applicability concerns | | |
| --- | --- | --- | --- | --- | --- | --- | --- |
| **Reference** | **Patient selection** | **Index test** | **Reference Standard** | **Flow and timing** | **Patient selection** | **Index test** | **Reference standard** |
| Hayashi et al. 2022 (14) |  |  |  |  |  |  |  |
| Duron et al. 2021 (15) |  |  |  |  |  |  |  |
| Bousson et al. 2023 (16) |  |  |  |  |  |  |  |
| Guermazi et al. 2022 (17) |  |  |  |  |  |  |  |
| Dell'Aria et al. 2024 (18) |  |  |  |  |  |  |  |
| Cohen et al. 2023 (19) |  |  |  |  |  |  |  |
| Russe et al. 2024 (20) |  |  |  |  |  |  |  |
| Ramadanov et al. 2025 (21) |  |  |  |  |  |  |  |
| Bachmann et al. 2024 (22) |  |  |  |  |  |  |  |
| Ziegner et al. 2025 (23) |  |  |  |  |  |  |  |
| Dupuis et al. 2022 (24) |  |  |  |  |  |  |  |
| Diaz et al. 2025 (25) |  |  |  |  |  |  |  |
| Lee et al. 2023 (26) |  |  |  |  |  |  |  |

**Supplementary Table 1:** Commercial AI tools for fracture detection.

|  | Risk of Bias | | | | Applicability concerns | | |
| --- | --- | --- | --- | --- | --- | --- | --- |
| **Reference** | **Patient Selection** | **Index test** | **Reference standard** | **Flow and timing** | **Patient selection** | **Index test** | **Reference standard** |
| Ye et al. 2025 (27) |  |  |  |  |  |  |  |
| Kim et al. 2023 (28) |  |  |  |  |  |  |  |
| Alzubaidi et al. 2024 (29) |  |  |  |  |  |  |  |
| Magneli et al. 2023 (30) |  |  |  |  |  |  |  |
| Breu et al. 2024 (31) |  |  |  |  |  |  |  |
| Yang et al. 2024 (32) |  |  |  |  |  |  |  |
| Anttila et al. 2023 (33) |  |  |  |  |  |  |  |
| Langerhuizen et al. 2020 (34) |  |  |  |  |  |  |  |
| Kitamura et al. 2019 (35) |  |  |  |  |  |  |  |
| Axenhus et al. 2025 (36) |  |  |  |  |  |  |  |
| Choi et al. 2020 (37) |  |  |  |  |  |  |  |
| Chung et al. 2018 (38) |  |  |  |  |  |  |  |
| Kekatpure et al. 2024 (39) |  |  |  |  |  |  |  |
| Ozkaya et al. 2022 (40) |  |  |  |  |  |  |  |
| Hendrix et al. 2021 (41) |  |  |  |  |  |  |  |
| Kim et al. 2021 (42) |  |  |  |  |  |  |  |
| Li et al. 2024 (43) |  |  |  |  |  |  |  |
| Gan et al. 2024 (44) |  |  |  |  |  |  |  |
| Dasegowda et al. 2024 (45) |  |  |  |  |  |  |  |
| Aldhyani et al. 2024 (46) |  |  |  |  |  |  |  |
| Wei et al. 2025 (47) |  |  |  |  |  |  |  |
| Binh et al. 2024 (48) |  |  |  |  |  |  |  |
| Ahmed et al. 2024 (49) |  |  |  |  |  |  |  |
| Ashkani-Esfahani et al. 2022 (50) |  |  |  |  |  |  |  |
| Kyung et al. 2024 (51) |  |  |  |  |  |  |  |
| Cheng et al. 2023 (52) |  |  |  |  |  |  |  |
| Yoon et al. 2021 (53) |  |  |  |  |  |  |  |
| Rashid et al. 2023 (54) |  |  |  |  |  |  |  |
| Jones et al. 2020 (55) |  |  |  |  |  |  |  |
| Yu et al. 2025 (56) |  |  |  |  |  |  |  |
| Ureten et al. 2022 (57) |  |  |  |  |  |  |  |
| Bluthgen et al. 2020 (58) |  |  |  |  |  |  |  |
| Franco et al. 2024 (59) |  |  |  |  |  |  |  |
| Zech et al. 2024 (60) |  |  |  |  |  |  |  |
| Zech et al. 2023 (61) |  |  |  |  |  |  |  |
| Gan et al. 2019 (62) |  |  |  |  |  |  |  |
| Zhang et al. 2023 (63) |  |  |  |  |  |  |  |
| Thian et al. 2019 (64) |  |  |  |  |  |  |  |
| Li et al. 2023 (65) |  |  |  |  |  |  |  |

**Supplementary Table 2:** Researcher-developed models for X-ray interpretation.

|  | Risk of Bias | | | | Applicability concerns | | |
| --- | --- | --- | --- | --- | --- | --- | --- |
| **Reference** | **Patient selection** | **Index test** | **Reference Standard** | **Flow and timing** | **Patient selection** | **Index test** | **Reference standard** |
| Olthof et al. 2021 (66) |  |  |  |  |  |  |  |
| Zech et al. 2023 (67) |  |  |  |  |  |  |  |

**Supplementary Table 3:** NLP / clinical-text and multimodal AI interpretation

Low concern

Unclear

High concern
